# Transcription-induced supercoiling explains formation of self-interacting chromatin domains in *S. pombe*

**DOI:** 10.1093/nar/gkx716

**Published:** 2017-08-11

**Authors:** Fabrizio Benedetti, Dusan Racko, Julien Dorier, Yannis Burnier, Andrzej Stasiak

**Affiliations:** 1 Center for Integrative Genomics, University of Lausanne, 1015 Lausanne, Switzerland; 2 Vital-IT, SIB Swiss Institute of Bioinformatics, 1015 Lausanne, Switzerland; 3 SIB Swiss Institute of Bioinformatics, 1015 Lausanne, Switzerland; 4 Polymer Institute of the Slovak Academy of Sciences, 842 36 Bratislava, Slovakia; 5 Institute of Theoretical Physics, École Polytechnique Fédérale de Lausanne (EPFL), 1015 Lausanne, Switzerland

## Abstract

The question of how self-interacting chromatin domains in interphase chromosomes are structured and generated dominates current discussions on eukaryotic chromosomes. Numerical simulations using standard polymer models have been helpful in testing the validity of various models of chromosome organization. Experimental contact maps can be compared with simulated contact maps and thus verify how good is the model. With increasing resolution of experimental contact maps, it became apparent though that active processes need to be introduced into models to recapitulate the experimental data. Since transcribing RNA polymerases are very strong molecular motors that induce axial rotation of transcribed DNA, we present here models that include such rotational motors. We also include into our models swivels and sites for intersegmental passages that account for action of DNA topoisomerases releasing torsional stress. Using these elements in our models, we show that transcription-induced supercoiling generated in the regions with divergent-transcription and supercoiling relaxation occurring between these regions are sufficient to explain formation of self-interacting chromatin domains in chromosomes of fission yeast (*S. pombe*).

## INTRODUCTION

Interphase chromosomes of higher eukaryotes are composed of linear arrays of self-interacting regions known as topologically associated domains (TADs) (1–7). The popularization of TADs concept in current literature was thanks to rapid development of chromatin conformation capture methods such as Hi-C that provide the information about spatial proximity of all chromosomal regions with respect to each other (8). The structure and the mechanism of formation of TADs are not yet firmly established and are the subject of intensive research (6,7,9). It is established though that for a given genomic distance, the contacts between chromosomal loci located in the same TAD are, on average, 2–3 times more frequent than between loci located in neighbouring TADs (1,2,10). The first studies of TADs in mice and drosophila, estimated TADs average size to be of about 1 Mb (1,2). Newer studies using higher resolution data revealed, however, a finer structure of TADs with an average size of about 200 kb (4). Lower eukaryotes, such as fission yeast (*Schizosaccharomyces pombe*), also have their interphase chromosomes composed of 50–100 kb-long TAD-like self-interacting domains (11). Since bacterial chromosomes also show TAD-like organization (12), it seems that organization of chromosomes into self-interacting domains is one of the unifying principles in biology.

In higher eukaryotes, TADs are thought to constitute regulatory compartments in which the increased frequency of intra TADs contacts ensures that cis-acting regulatory elements, such as enhancers and promoters, interact only or almost only with their legitimate partners (6,13). As a corollary, legitimate enhancer-promoter partners are normally located in the same TAD even if their genomic separation is as large as 1 Mb (6). Although we seem to understand the role of TADs in gene regulation, we still do not understand how TADs are organized and what are the physical effects and biological mechanisms that lead to their formation.

Detection of self-interacting domains in chromosomes of *S. pombe* offered a possibility to study TADs formation in lower eukaryotes. This may be illuminating as *S. pombe* does not have CTCF protein that is known to be implicated in the organization of TADs in higher eukaryotes. Interestingly, the genes located in individual self-interacting chromatin domains in *S. pombe* are oriented in such a way that direction of transcription points outwards from the centre of each domain so that domains boundaries are flanked by genes in a convergent orientation (11). Starting with this observation, we build models that take into account torque introduced by transcribing RNA polymerases in these specific orientations within *S. pombe* TADs. Our models also account for recent proposition that positive supercoiling is preferentially relaxed by type I DNA topoisomerase associated with elongating RNA polymerases (14) and that type II DNA topoisomerases were shown to be localized at TADs borders (15). By comparing simulated chromosomal contact maps with experimental chromosomal contact maps, we can estimate the extent of transcription-induced supercoiling needed to recapitulate the experimental data. We test our model against other models and also consider possible role of supercoiling in TADs of higher eukaryotes.

## MATERIALS AND METHODS

Molecular dynamics simulations were performed using the general-purpose particle simulation toolkit HOOMD-blue [http://codeblue.umich.edu/hoomd-blue] (16–18) and were run on GPUs. Ten nanometre chromatin fibres were modelled as semi-flexible beaded chains. The diameter of each bead was assumed to correspond to 10 nm and it set the Lennard–Jones length unit (σ_L–J_). Since the linear density of 10 nm chromatin fibres is of ∼400 bp/10 nm (19) one bead in our model is assumed to correspond to 400 bp. All simulations were performed under periodic boundary conditions and in most of the cases included five independent copies of the modelled systems. The conversion between the simulation time step and the corresponding physical time step was done using Stokes’ approximation (20). This conversion depends on the viscosity of solution for which the simulation is done. For solutions with viscosity of water, 1 simulation time step corresponds to ∼5 μs of physical time. However, within chromosomal territories the viscosity can by even 15 000 higher than this of water (21) and in such a case one simulation time step would correspond to ∼75 ms.

A cut and shifted Lennard–Jones potential (repulsive part only) with *r*_cut_ = 1σ_L–J_ set the excluded volume interactions between individual beads. Our model, in addition to standard harmonic bonding potential, also included bending and torsional potential. The energy unit ε_0_ corresponded to 1*k*_B_*T*. The bending stiffness was set to ε_b_ = 5ε_0_, giving the persistence length corresponding to ∼50 nm, which is within the range of experimentally measured persistence length of chromatin fibres that can range from 30 nm (22) to 150 nm (23). The torsional stiffness was based on the potential minimizing dihedral angles, as earlier described (24). The harmonic dihedral potential had the form *V*(ϕ) = 0.5*k*(ϕ^2^) where the angle ϕ ranged from –π to π and *k* was set to 4ε_0_. The ratio between bending and torsional resistance of our modelled chromatin fibres was such that when they were supercoiled ∼70% of Δ*Lk* was converted into Δ*Wr*. Auto-correlation times of respective simulated systems were estimated by following changes of their radius of gyration (10).

In addition to standard portions of chromatin fibres, which we modelled as described above, we also included ‘models’ of RNA polymerases and DNA topoisomerases. Since we were only interested in the transcription induced supercoiling and not in the synthesized RNA chains, we modelled RNA polymerases as rotational motors inducing axial rotation of modelled chromatin fibres. We could set the direction of rotation and the torque values acting on beads forming the motors. The tested torque values ranged from 1 to 4 pN·nm. The majority of simulations were performed with the strength of torsional motors set to 2 pN·nm. If the torque values were different they were indicated on the corresponding figures.

To model action of topoisomerases that permit passive torsional relaxation of chromatin fragments as well as passive disentanglement resulting from intersegmental passages we included into our models swivels and zones of facilitated passages. Swivels consisted of inter-beads bonds, in which the dihedral potential was set to 0. The zones of facilitated passages consisted of stretches of several beads, which showed very weak excluded volume potentials. This weak excluded volume potential permitted thermally driven intersegmental passages but maintained the torsional resistance of modelled chromosome fragments. The zones of facilitated passages were arbitrarily set to 10 beads to make intersegmental passages more likely to happen. This was to compensate for the simplicity of our model where we do not have topoisomerases bound at some specific sites of chromatin and attracting other segments for a possible passage.

## RESULTS

### Effects of divergent transcription in a 50 kb-long chromatin loop

Since the early studies of Liu and Wang (25,26) it is known that transcribing RNA polymerases exert torque on the transcribed DNA molecules, which causes axial rotation of the transcribed DNA. Since in a dense cellular environment, the torque acting on transcribed DNA is opposed by large hydrodynamic drag, one observes generation of torsional stress in transcribed DNA molecules. As a consequence of the right-handed structure of DNA double helix, the torque generated during transcription produces torsional stress that causes formation of positive DNA supercoiling ahead of transcribing polymerases and of negative DNA supercoiling behind transcribing RNA polymerases (25,26). When two or more RNA polymerases directly follow each other, the negative torsional stress generated by the leading polymerase is cancelled out by the positive torsional stress generated by the next RNA polymerase. More complex situation occurs though in DNA/chromatin regions with divergent or convergent transcription. The torsional stress generated by a RNA polymerase moving in one direction adds to the torsional stress generated by a RNA polymerase moving in the opposite direction. If the accumulated torsional stress is not relaxed by DNA topoisomerases, DNA regions between oppositely oriented RNA polymerases, which transcribe different strands of the same DNA molecule, react to accumulated torsional stress by forming supercoils (25–27).

**Figure 1.**
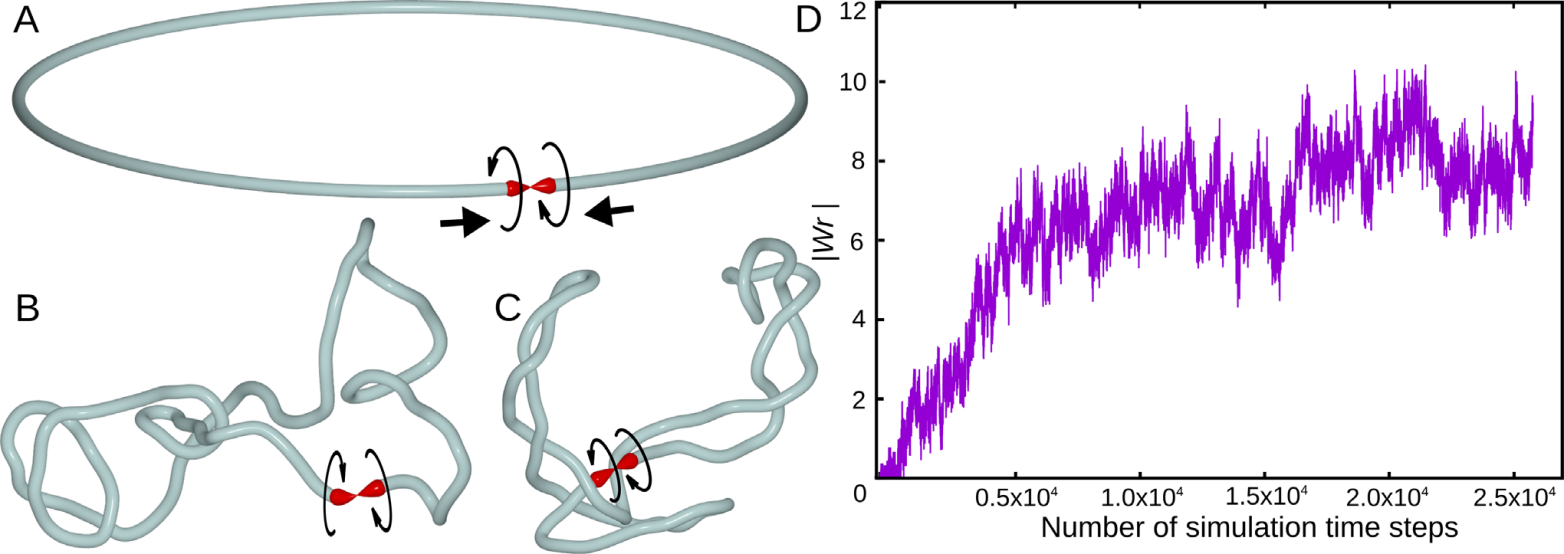
Simulations of effects of divergent transcription in a small chromatin loop in which positive supercoiling generated ahead of RNA polymerases is dissipated. (**A–C**) Simulation snapshots showing gradual accumulation of negative supercoiling. The starting configuration is shown in A. The arrowhead-shaped objects indicate positions of torsional motors that mimic the effect of RNA polymerases by generating torque and inducing local axial rotation of the chain. The modelled chain reflects the properties of semi-flexible polymers like DNA or chromatin fibres with bending and torsional resistance. Only in the short region between the two torsional motors the chain has no torsional resistance. The region with no torsional resistance (swivel) is graphically presented as a sharp tip contacting a flat surface to resemble a tip of a spinning top that is in a contact with the supporting surface but is free to rotate. (**D**) The magnitude of writhe (one of the measures of supercoiling (32)) increases and then saturates with time (the writhe in negatively supercoiled DNA and chromatin fibres is negative).

**Figure 2.**
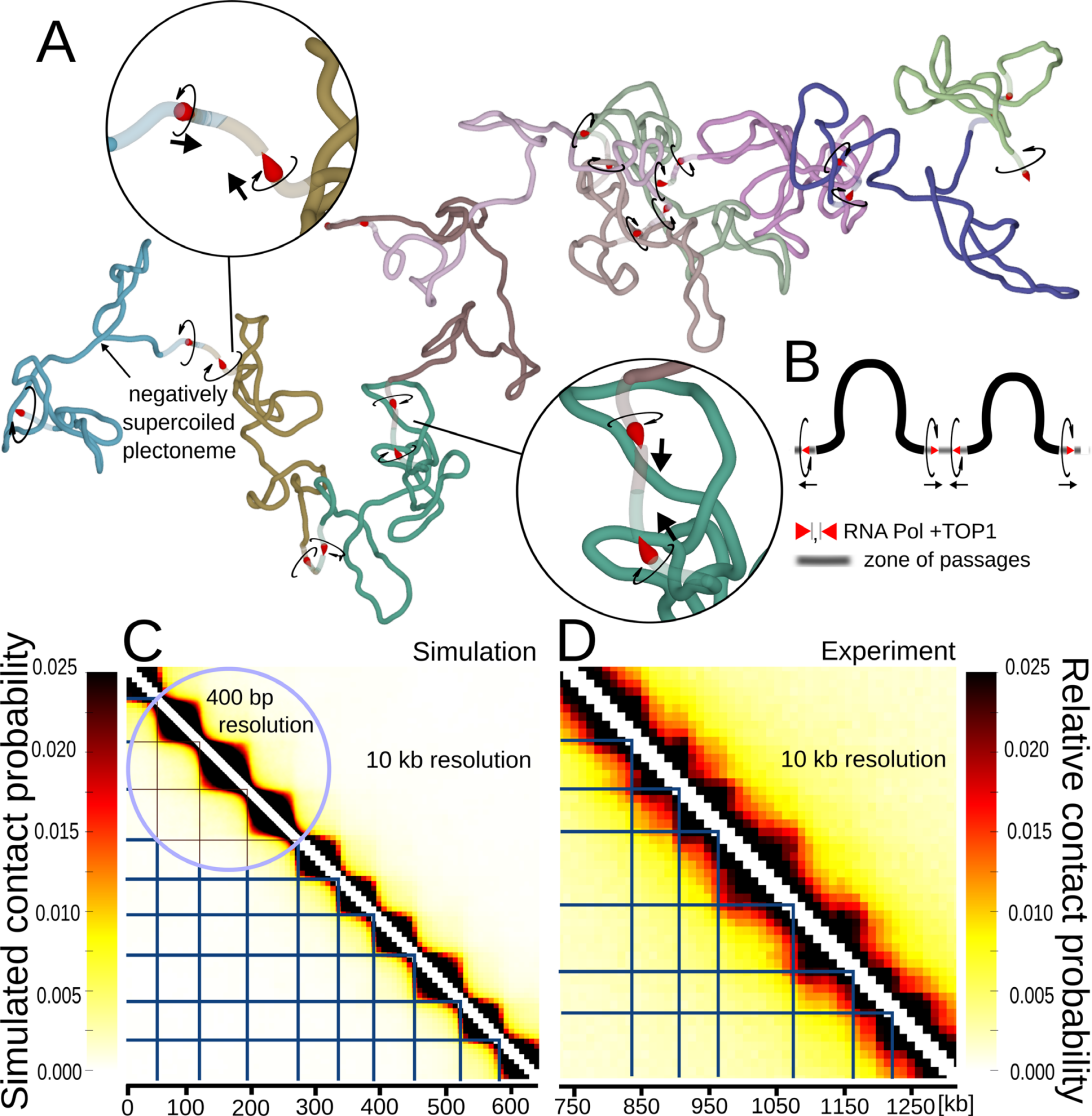
Simulations of large chromosome fragments with 10 divergent transcription domains separated by sites where torsional stress gets dissipated and zones where portions of modelled chains can pass through each other. (**A**) Simulation snapshot. Each domain with divergent transcription is marked with a different colour. Torsional motors and swivel sites are presented as in Figure 1A–C. The zones where portions of the chain can pass through each other are presented as semi-transparent. (**B**) Schematic linear map of two consecutive domains showing the location of modelled RNA polymerases with TOP1 preceding them and also showing location of zones of passages. Circular arrows indicate the direction of rotation induced by respective polymerases. Normal arrows indicate the direction of transcription. (**C**) Contact map obtained upon analysis of nearly 20 millions of configurations of modelled chromosome fragments such as shown in A. Notice that each of the modelled divergent transcription domains forms a TAD-like region with increased frequency of internal contacts. The blue horizontal and vertical lines indicate positions of gene convergence imposed in our model. (**D**) Experimental contact map of a portion of *S. pombe* chromosome with 10 divergent transcription domains. This contact map was obtained using experimental data deposited by Mizuguchi *et al.* (11) and corresponds to the portion of chromosome 2 ranging from ∼750 to 1300 kb positions of that chromosome. The blue horizontal and vertical lines indicate positions of local maxima of gene convergence in the corresponding chromosome fragment as determined by Mizuguchi *et al.* (11). Notice that the sizes of self-interacting domains in the modelled chromosome fragment (A and C), reflect the distribution of self-interacting domain sizes observed in all *S. pombe* chromosomes and were not adjusted to fit the sizes of self-interacting domains in the chromosome fragment, whose contact map is shown in C.

To simulate the effect of divergent transcription on chromatin fibres, we introduced into our models torsional motors that locally exert a torque on semi-elastic chains with bending and torsional resistance of chromatin fibres (see Materials and Methods). Our models are dynamic and supercoiling introduced by torsional motors can freely diffuse along the modelled chromatin fibres. We have set the strength of torsional motors to 2pN·nm because this value is within the physiological range and it roughly corresponds to half of the maximal torque that can be exerted on chromatin by a transcribing RNA polymerase (28). To illustrate the principle of transcription induced supercoiling, we present first the behaviour of one divergently transcribed region, with the length corresponding to ∼50 kb long chromatin fibre, which is a typical size of self-interacting domains in *S. pombe*. For purpose of demonstration we have closed that modelled divergently transcribed region into a loop (see Figure 1A). The closure point is flanked by two torsional motors which we present in Figure 1A as arrowheads whose direction corresponds to the direction of transcription of two RNA polymerases diverging from the transcribed region and thus converging towards the closure point. The torsional motors introduce torque acting in opposite directions, as indicated with circular arrows. These opposing directions of rotations correspond to the directions of DNA rotation generated by two RNA polymerases diverging from the transcribed region and thus converging towards the swivel point (directions of transcriptions are indicated with thick arrows). Between the two motors we placed a swivel that permits the relaxation of torsional stress in a manner similar to the action of type I DNA topoisomerases (29) but also recapitulating the action of type II DNA topoisomerases that efficiently relax torsional stress in chromatin by a localized action involving passages between incoming and outgoing DNA linkers of the same nucleosome (30). The swivel is presented as a zone where two sharp tips of torsional motors approach each other. Since there is a swivel between the two motors, the positive supercoiling generated ahead of the RNA polymerases can freely dissipate, as proposed by Baranello *et al.* (14). However, the negative supercoiling, that normally is generated behind RNA polymerases (25–27,31), cannot dissipate in this model (supercoiling can partially dissipate though in the model presented in Figure 2). This setting of the model corresponds to the biological situation where negative supercoiling is long lived, whereas the positive supercoiling is rapidly eliminated by the action of DNA topoisomerases associated with RNA polymerases (14).

Figure 1 shows how our model evolves with time. For the purpose of didactic exposition, we start with nearly perfect circular configuration (Figure 1A). Once motors have started, modelled chromatin fibre progressively supercoils (Figure 1B) until reaching a steady state level of negative supercoiling (Figure 1C). Figure 1D shows how the magnitude of writhe, which is a measure of supercoiling (32), initially increases and then reaches a steady state. The steady state of writhe in this model results from stalling of torsional motors, which occurs when the accumulated torsional stress opposing the action of torsional motors becomes as large as the strength of the motors. Similar stalling of RNA polymerase by high level of transcription-induced negative supercoiling was observed in single molecule studies performed *in vitro* (31). In living cells the extent of supercoiling generated by ongoing transcription is also expected to reach a steady state level (27). However, this is not achieved by permanent stalling of RNA polymerases but by the action of DNA topoisomerases that prevent the accumulation of too strong torsional stress in the DNA (33). In living cells keeping negative torsional stress to moderate levels facilitates separation of DNA strands needed for the initiation of transcription (34,35), whereas too strong levels of negative supercoiling cause such undesirable consequences as excessive DNA denaturation (36) or formation of stable R-loops (37). The model presented in Figure 1 is just to explain the action of torsional motors and it serves only as an introduction to a more advanced model presented in Figure 2, which we use to explain formation of self-interacting domains in *S. pombe*.

### Modelling of large portions of *S. pombe* chromosome having 10 domains with divergent directions of transcription

Next, we used our model with torsional motors reproducing effects of transcription-induced supercoiling to simulate relatively large linear chromosome fragments of *S. pombe* chromosomes. We therefore constructed linear models composed of 10 regions with divergent transcription. Each such region has at its ends oppositely oriented torsional motors generating torque in analogous ways to RNA polymerases (see Figure 2A and B). Each RNA polymerase is presented in Figure 2A and B as an arrowhead shaped object pointing in the direction of transcription. The directions of rotation induced by individual RNA polymerases are indicated with circular arrows. Although there are usually several genes per self-interacting domain in *S. pombe*, several RNA polymerases moving in the same direction introduce the same number of rotation of the template as one RNA polymerase. Therefore, one torsional motor per direction can ‘replace’ several RNA polymerases. In our model, motors are not moving along the modelled chromatin fibre. However, as our models are coarse-grained and each motor occupies two beads, which correspond to 800 bp, the motors can be considered as representing RNA polymerases moving within 800 bp region and thus able to introduce ∼80 rotations just by acting where they are in the model.

To reflect the observation that TOP1 is associated with elongating RNA polymerases in such a way that it can relax torsional stress encountered before but not after transcribing polymerase (14), we introduced swivels ahead of each modelled RNA polymerase. The swivels are presented as places where leading tips of RNA polymerases contact preceding segments of modelled chromatin fibres (see Figure 2A and B). To account for the action of type II DNA topoisomerases, which catalyse passages of double-stranded DNA regions through each other, we placed zones with greatly decreased excluded-volume potential at every border between divergently transcribed regions. In fact, recent studies of chromosomes in higher eukaryotes revealed that type II DNA topoisomerases localize at borders of TADs (15). It is not known yet, though, whether in *S. pombe* topo II localizes at borders of self-interacting chromatin domains. In Figure 2A and B, these zones are shown as semi-transparent portions of the modelled chromosome fragment. Thermally driven collisions with other fragments occurring at these zones are often sufficiently strong to overcome the weak excluded-volume potential and let other portions of simulated chromosomes to pass through. The torsional resistance of modelled chromosome fragments was kept unchanged in the semi-transparent zones. Figure 2B schematically indicates the positions of RNA polymerases with associated TOP1 and of zones of passages in two sequential domains. Directions of rotation introduced by respective RNA polymerases are indicated together with the directions of transcription which would introduce these rotations.

Figure 2A shows a snapshot of a simulated chromosome fragment having ∼600 kb in length and containing 10 domains with divergent directions of transcription. Each domain is shown in a different colour. The snapshot was obtained after a steady state equilibrium was reached, i.e. after radius of gyration of the entire construct got stabilized. The snapshot shows that individual regions with divergent directions of transcription are self-compacted due to supercoiling. Some domains are forming negatively supercoiled plectonemes, whereas other domains are more disordered. Insets with magnified views show borders between domains with torsional motors, swivels and the regions where the modelled chains can pass through each other (indicated as semi-transparent zones). Figure 2C shows a contact map generated using nearly 20 million configurations obtained for 5 independently evolving replicas of the modelled chromosome fragment, where each replica was pre-thermalized and then evolved for more than 8 correlation times. The contact map clearly shows that individual regions with divergent transcription form self-interacting domains that manifest themselves as triangle-shaped regions in the contact map. The simulated contact map is shown at two different resolutions. The 400 bp resolution (encircled region) naturally arises from the contact scoring in the modelled beaded chain where every bead corresponds to 400 bp. The 10 kb resolution corresponds to the resolution of Hi-C experiments and in simulations this resolution is obtained by averaging over sequential groups of 25 beads.

Figure 2D shows the experimental, Hi-C contact map of ∼600 kb-long fragment of *S. pombe* chromosome with 10 divergently transcribed domains. The contacts in the same chromosome fragment were analysed by Mizuguchi *et al.* in their Figure 1f (11) and our Figure 2D was generated using the data deposited by Mizuguchi *et al.*

Before comparing the modelled and experimental contact maps, it should be mentioned that the sizes of self-interacting domains in our model (Figure 2A and C) were just randomly sampled from the distribution of experimentally determined domain sizes in *S. pombe* chromosomes. Our modelled domains were not adjusted to fit the sizes of self-interacting domains analysed in the experimental contact map shown in Figure 2D. Despite these differences in domain sizes, the simulated and experimental contact maps (both at 10 kb resolution) qualitatively resemble each other. Both maps show characteristic triangles indicating regions with increased frequencies of internal contacts. These regions correspond to modelled or real domains with divergent directions of transcription. With the strength of torsional motors set to 2 pN·nm, which corresponds to ∼50% of the RNA polymerase stalling torque (28), the supercoiling in modelled self-interacting domains may be weaker than achievable *in vivo*. To roughly evaluate what level of supercoiling may correspond to the situation *in vivo*, we have performed additional simulations. We modelled the same chromosome fragment with 10 divergently transcribed domains but with different settings of the strength of torsional motors. In Supplementary Figure S1, we show contact maps obtained in simulations where the strength of all torsional motors was set to 0, 1, 2 or 4 pN·nm, respectively. Visual evaluation of contact maps, including such elements as the ratio of interactions within and between neighbouring domains and shapes of TADs on the contact maps, suggests that out of tried torsional strengths of motors the 2 pN·nm is already sufficiently strong to reproduce the experimental data. We also measured the writhe of individual modelled domains resulting from the different strengths of torsional motors. On average the writhe value calculated for 50 kb large chromosomal domain flanked by two motors amounted to –1.7, –3.5 and –6.1 for the torsional motor strengths of 1, 2 and 4 pN·nm, respectively. Therefore, our results suggest that the writhe within TADs is weak as it amounts to only ∼3–4 negative superturns per 50 kb-large chromatin domain.

In Figure 2C, we indicated with blue vertical and horizontal lines the positions corresponding to regions where modelled transcribing RNA polymerases converge. In Figure 2D, we used blue vertical and horizontal lines to indicate positions of local maxima of gene convergence. The local maxima values of gene convergence are based on the analysis of gene convergence profiles presented by Mizuguchi *et al.* (11) in their Figure 1f. It is important to add here that due to a large genomic window over which gene convergence was scored by Mizuguchi *et al.* (11), smaller regions of gene convergence are visible as local maxima in gene convergence profile but may still have overall negative value. Looking at Figure 2C and D it is striking how well the modelled and experimentally defined borders of *S. pombe* TADs coincide with modelled and experimentally defined regions of gene convergence.

### Simple modelled system with stallable torsional motors and localized regions for intersegmental passages behave as a homeostatic control system of supercoiling level

**Figure 3.**
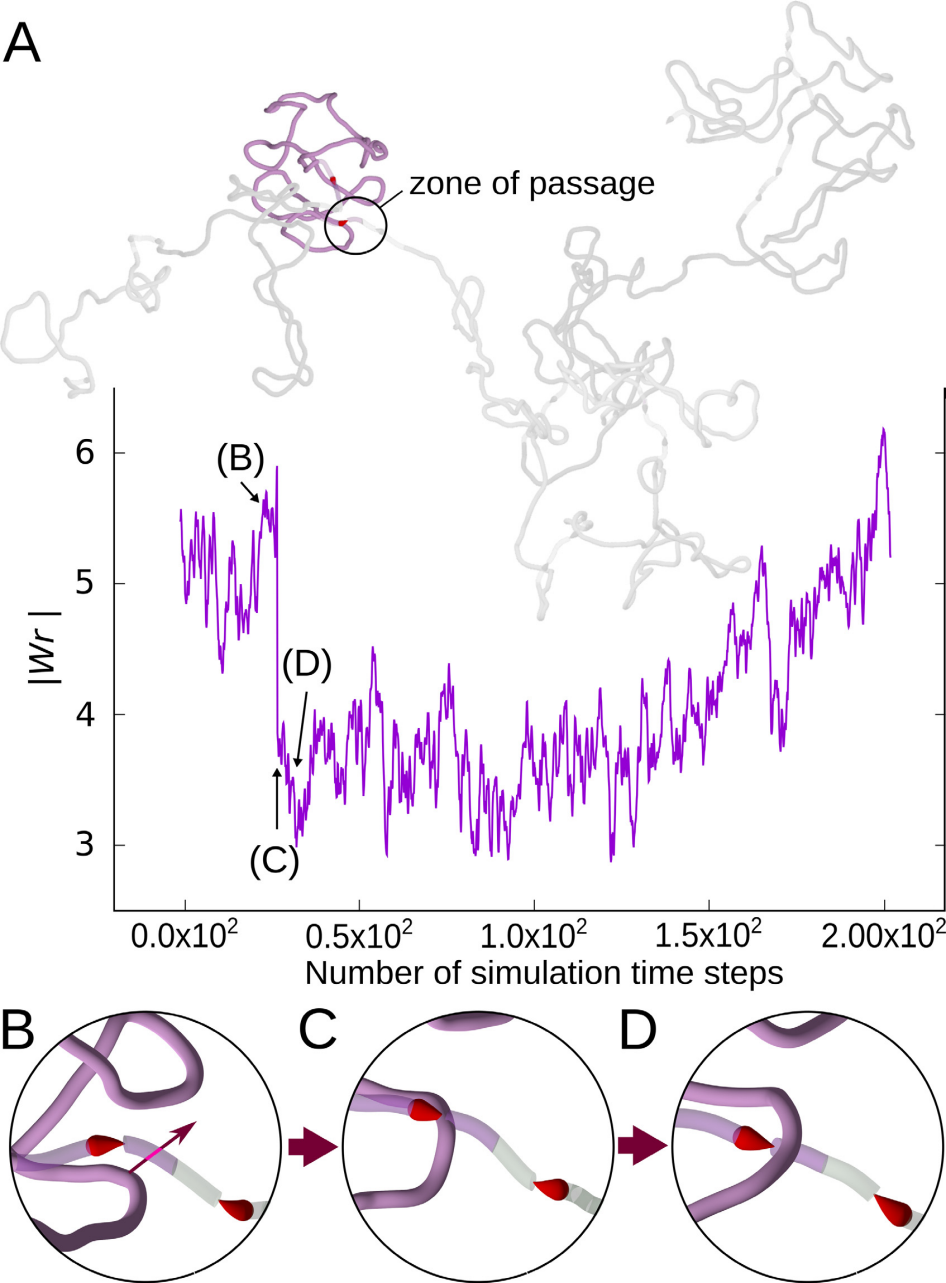
Homeostatic control of supercoiling. (**A**) Evolution of writhe in one domain (highlighted in blue) within thermally equilibrated chromosome fragment with 10 domains. The snapshot of the entire chromosome fragment is taken at the beginning of the simulation interval for which the writhe profile is shown. After about 25 simulation steps during which writhe was changing moderately there was a simulation step time during which writhe has changed by ∼2. Such a change is an indication of an intersegmental passage. (**B–D**) Snapshots of the zone of passage (encircled in A), showing the progress of one intersegmental passage. Simulation time steps corresponding to these snapshots are indicated with arrows in the writhe profile. The writhe profile shows that after the initial drop of the magnitude of writhe after the passage, the writhe values tend to return to the level before the passage.

We investigated next whether our simulated systems behave as ‘static’ systems, where motors are simply stalled by the accumulated torsional tension or whether they show a more complex behaviour with ongoing topoII-like passages, partially relaxing torsional stress and torsional motors re-establishing then the original level of supercoiling. To this aim, we have performed additional simulations and traced the evolution of writhe of every domain. We observed characteristic very sudden changes of writhe by about two, indicating that they are due to intersegmental passages (see Figure 3). We verified by direct observations of corresponding snapshots taken before and after the writhe change that these events indeed corresponded to intramolecular passages involving semi-transparent and non-transparent regions of the same simulated domain (see Figure 3). Video S1 shows the passage event more clearly. The frequency of such intersegmental passages was irregular but on average in individual domains they were occurring with the rate of ∼600 events/s, if our modelled system was evolving in water. Figure 3 also shows that after a passage and resulting partial relaxation, the supercoiling level in the affected domain is progressively re-established. After a passage that decreases the magnitude of writhe, the torsional stress in modelled chromatin decreases, permitting stalled torsional motors to restart and to re-establish the original level of supercoiling. Therefore, our simulated system with torsional motors, swivels and modelled TOP2 behaves as a homeostatic system that re-establishes a given level of supercoiling after a partial relaxation of torsional stress by topoisomerases. That simple homeostatic system could be regulated to different levels by several independent parameters. These parameters are the speed of transcription, which in turn can be regulated by multitude of mechanisms, the efficiency of the swivels, i.e. of TOP1 acting ahead of RNA polymerases and the activity of type II DNA topoisomerases, which in the model setting could be regulated by changing the size of zones where passages can happen.

### Generic polymer models with cohesin rings placed at TADs borders are unable to recapitulate the Hi-C data

The results presented in Figure 2 support our proposal that transcription-induced supercoiling is responsible for the formation of self-interacting domains in chromosomes of *S. pombe*. However, Mizuguchi *et al.*, in their paper showing that S. pombe chromosomes are composed of linear arrays of TADs-like chromatin domains, have proposed another possible mechanism of their formation (11). According to that proposal the mere presence of such large macromolecular structures like cohesin rings encircling chromatin fibres and placed always at specific sites could be sufficient to create TADs-like domains (see Figure 3f in (11)). If contacts between chromatin portions separated by cohesin rings are disfavoured, then for the same genomic distance the frequency of contacts between a pair of loci not separated by a cohesin ring should be higher than between loci separated by such a ring.

**Figure 4.**
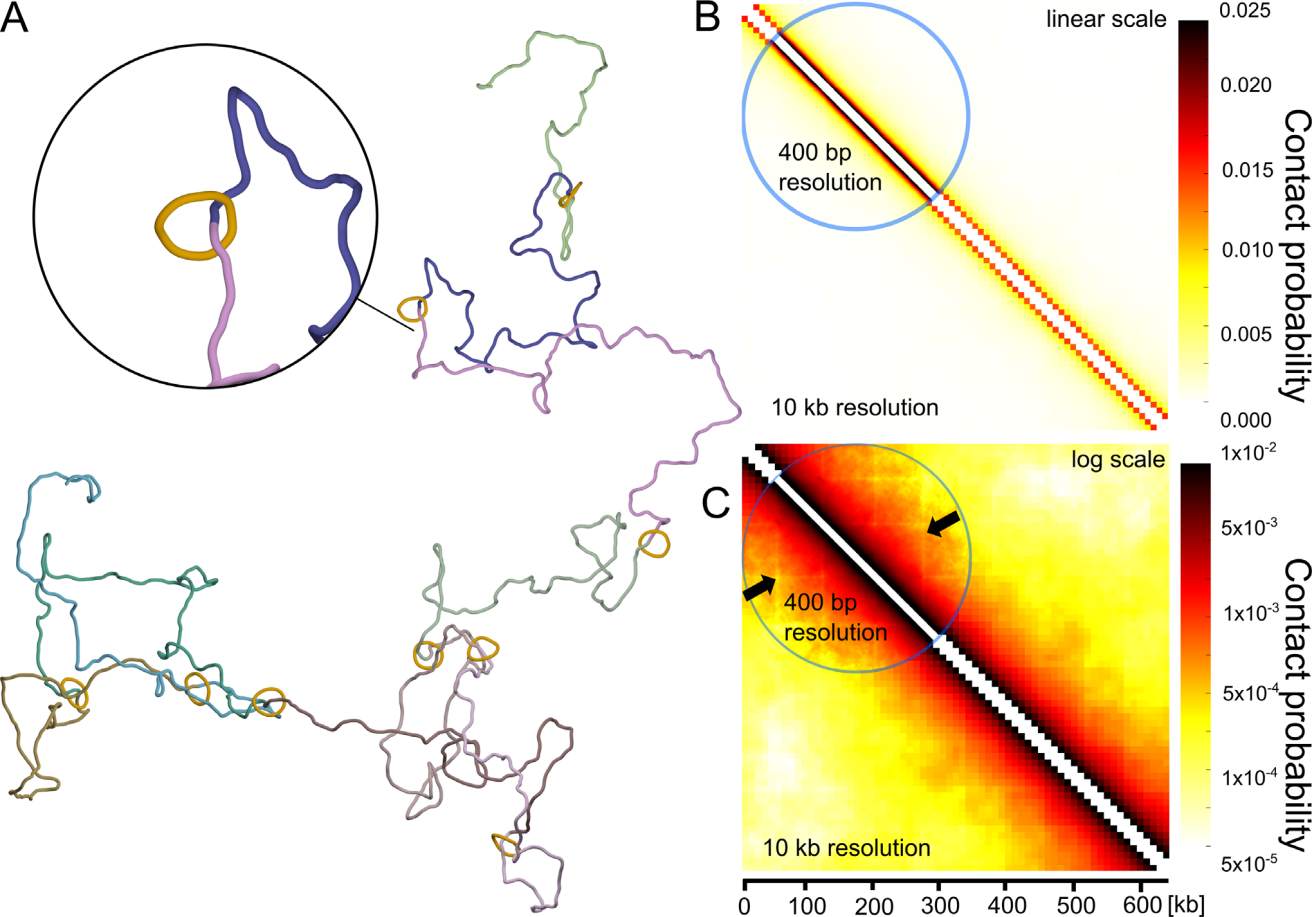
Simulations testing the cohesin barrier model. (**A**) Snapshot from the simulation of chromatin fibres with encircling cohesin rings maintained at regular intervals. (**B** and **C**) Contact maps obtained for a large statistical sample of equilibrated configurations such as shown in A. In B, the applied colour scale is linear as the one used in the experimental contact maps of Mizuguchi *et al.* (11) and in the simulated contact maps presented in Figure 2. In C, the applied colour scale is logarithmic. Notice that the presence of localized cohesin rings results in a local depletion of contacts at chromatin portions shielded by the rings, but does not result in formation of characteristic triangles indicating presence of self-interacting domains. The arrows indicate lines with depleted contacts.

We decided to test by simulations the consequences of the mere physical presence of large rings encircling modelled chromatin fibres. Cohesin rings, modelled as circles composed of 16 beads (each with 10 nm diameter) and forming rigid rings with overall diameter corresponding to 50 nm, were tethered to modelled chromosome fragment at the average genomic separation corresponding to ∼50 kb. Subsequently, the modelled chromatin fibres with the attached cohesin rings were subject to random thermal fluctuation until equilibrium. It is important to add here that in contrast to the previous models, presented in Figures 1 and 2, in the ‘cohesin barrier’ model, there were no torsional motors mimicking the action of RNA polymerases, no swivels and no passage zones mimicking the action of DNA topoisomerases. Figure 4A shows a snapshot from simulations of chromatin fibre having cohesin rings tethered at specific sites. Visual analysis of this configuration and of many other snapshots from these simulations does not seem to reveal self-interacting domains induced by the mere presence of cohesin rings. Figure 4B and C shows, using two different colour scales, the contact maps obtained for the simulated configurations. When we applied the same linear colour scale as used for the contact maps presented in Figure 2, the contact map obtained for the ‘cohesin barrier’ model was essentially featureless (see Figure 4B). There were no characteristic triangles indicating the formation of self-interacting domains. After applying logarithmic colour scale to the contact map (Figure 4C) it is visible though that the sites where modelled cohesin rings are located are in fact depleted of contacts. The lines with depleted contacts (indicated with arrows) are more visible in the portion of the contact map shown with 400 bp resolution (see also Supplementary Figure S2 for a magnified image of a section of such a contact map). However, this local contact depletion does not lead to formation of self-interacting domains.

The simulation results presented above speak against the model proposed by Mizuguchi *et al.* (11), and postulating that a physical presence of a relatively large protein bound at specific genomic sites results in formation of TADs-like chromatin domain. Mizuguchi *et al.* (11) did not consider more complex models such as loop extrusion models (5,7) as these more complex models were proposed only for chromosomes of higher eukaryotes in which the interplay between CTCF-protein (absent in *S. pombe*) and cohesin may be important in stabilization of chromatin loops. Our model of transcription induced supercoiling, implicating action of topoisomerases at the borders of TADs, explains formation of TAD-like self-interacting chromatin domains in *S. pombe* without invoking rather complex mechanisms of chromatin loop extrusion (5,7).

## DISCUSSION

We have shown above that transcription-induced supercoiling together with the localized action of topoisomerases, is sufficient to explain the formation of self-interacting domains in the chromosomes of *S. pombe*. Our simulations suggest that transcription-induced supercoiling is the cause of a local increase in contact frequency. In addition, the sites of topoisomerase action, where torsional stress and entanglements can be released, are required for the formation of borders between regions with increased frequency of contacts. Can this mechanism be universal and also apply to chromosomes of higher eukaryotes, such as humans?

In contrast to *S. pombe*, with chromosome domains containing genes in divergent orientation and thus with a high potential to accumulate negative supercoiling, TADs in mammalian chromosomes are not known to consist of divergently transcribed genes. However, divergent orientations of genes are not necessary to generate supercoiling in chromatin. Recent studies have shown that type I topoisomerases associated with RNA polymerases have the ability to relax the positive supercoiling arising ahead of transcribing polymerases but not the negative supercoiling generated behind them (14). As a result, transcription generates net negative supercoiling (14). Importantly, just one transcribing RNA polymerase per TAD would be sufficient to supercoil an entire TAD as individual RNA polymerases could introduce as much as two supercoils per second (31,38) when molecular crowding and RNA polymerase interactions leading to formation of transcription factories (39) prevent RNA polymerases from encircling the transcribed chromatin fibre. Therefore, also in higher eukaryotes transcription can induce localized supercoiling (27,40,41) causing localized enhancement of contacts.

In fact, Naughton *et al.* (27) studying cultured human cells have shown that large portions of transcribed chromatin are negatively supercoiled despite being enriched in TOP1, that is known to relax torsional stress in chromatin. Baranello *et al.* have resolved this apparent paradox by proposing that interaction between RNAPII and TOPI positions the latter in such a way that it can relax torsional stress arising ahead but not behind transcribing RNA polymerases (14). As the consequence, promoters of active genes carry a sturdy level of negative supercoiling since promoters are upstream of RNAPII and are thus not accessible to TOPI but are affected by negative supercoiling generated upstream of advancing RNA polymerase (14). Along the gene bodies, TOP1 associated with RNAPII is active and can in principle relax positive or negative torsional stress encountered ahead of transcribing polymerases. As a consequence, the level of negative supercoiling in the gene bodies is lower than in promoter regions (14). However, as there is a wave of positive supercoiling ahead of each transcribing RNA polymerase, it is the positive supercoiling that is preferentially relaxed by TOP1 (14). Consequently, there is a moderate accumulation of negative supercoiling in the bodies of transcriptionally active genes (27). Naughton *et al.* also showed that both transcription and topoisomerase action are required for accumulation of negative supercoiling in transcribed chromatin portions (27). This gives additional support to the proposal that out of two twin supercoiled domains associated with every transcribing RNA polymerase, the positive domain is preferentially relaxed by topoisomerases and therefore transcription induces net negative supercoiling (27).

Studies of yeast cells also support the notion that their chromatin is negatively supercoiled. Bermudez *et al.* (42) showed that *in vivo* binding of psoralen to chromatin in yeast chromosomes increases with the genomic distance to telomeres. Since binding of psoralen to DNA increases with negative supercoiling and since in yeast the torsional stress can dissipate by rotation of chromosome ends, the authors concluded that there is an ongoing generation of negative supercoiling in yeast chromosomes (42).

Our model predicts that TADs in *S. pombe* would disappear when the transcription is shutdown. We are not aware of experiments testing the influence of transcription on TADs in *S. pombe*, however in bacteria TADs indeed disappear when transcription is completely inhibited by antibiotics (12).

Our modelling was testing the situation where there are at least two divergently transcribed genes in each TAD. As discussed above for the case of TADs in higher eukaryotes this condition may not be always required. In the viscous nuclear environment, it would be sufficient to have one RNA pol transcribing towards the TAD border. The negative supercoiling generated behind the polymerase would then slowly diffuse towards the other border and therefore induce supercoiling in the entire chromatin portion behind transcribing RNA polymerase and extending towards the supercoiling sink at the other border of the TAD. Our earlier simulations have shown that in viscous environment, torsional motors can supercoil DNA molecules even if these are linear or contain nicks (43).

What about the TAD borders in higher eukaryotes? Are they the regions where topoisomerases preferentially remove the torsional stress and can also perform intersegmental passages permitting disentanglement and spatial separation of neighbouring TADs? Very recent ChIP-Seq studies of mammalian chromosomes have shown that the type II topoisomerase Top2B interacts with CTCF protein and localizes at border elements of individual TADs (15).

Existence of long lived supercoiling contrasts, however, with a popular but probably incorrect notion that there is a pervasive, non-controlled action of type I and type II DNA topoisomerases in living cells. Such an action would rapidly relax torsional stress in DNA. However, a growing number of studies have shown that the action of DNA topoisomerases is highly regulated (33). In bacteria, for example, which keep their DNA negatively supercoiled and which have various type I and II DNA topoisomerases, there are interesting, but not completely understood mechanisms that protect negatively supercoiled DNA from relaxation. Bacterial type I DNA topoisomerases require their substrate DNA to be strongly negatively supercoiled and thus partially melted to be able to decrease its supercoiling level to the physiological one (44,45). Bacterial topoisomerase IV, which is type II topoisomerase and thus able to pass duplex DNA segments through transient openings in other duplex DNA segments, hardly acts on negatively supercoiled DNA molecules. However, topoisomerase IV is highly active in relaxing positively supercoiled DNA molecules (46,47). Presumably, the geometry of juxtapositions between contacting DNA segments are different in positively and negatively supercoiled DNA and this determines whether a passage can happen or not (47–49). In the case of higher eukaryotes, observations of chromosome territories with very little inter-chromosomal intermingling (50) have led to proposals that there are essentially no free passages between chromatin fibres (51,52). Hi-C studies showed that the decay rate of intra-chromosomal contacts with their genomic distance is only consistent with the situation where there are no free passages between chromatin fibres forming the same chromosome (8). Very recent studies started to reveal a complex regulation of type I and type II DNA topoisomerases in mammalian cells by their direct interactions with RNA polymerases (14), chromatin remodeller SMARCA4 (53), BAF complexes (54) or CTCF proteins (15).

## Supplementary Material

Supplementary Data
