# Supplementary material for: Transcription-induced supercoiling explains formation of self-interacting chromatin domains in S. pombe
Source: Nucleic Acids Res. 2017 Aug 11;45(17):9850–9. doi: 10.1093/nar/gkx716 (PMC5622301; doi:10.1093/nar/gkx716)
Supplement: Supplementary Data [file gkx716_supp.zip › nar-01875-n-2017-File006.pdf]

Figure S1.
Effects of the strength of torsional motors on the contact maps.
The contact maps correspond to systems that only differ in the strength of torsional motors (0, 1, 2 or 4pN·nm, respectively). At the highest strength of torsional motors one observes clearly defined zones of contacts between neighbouring domains. When supercoiling is strong, individual domains are strongly compacted and each bead in a given domain has a similar probability of contacting a given bead in the neighbouring domain.

Figure S2.
Physical presence of cohesin rings is not sufficient to generate formation of self-interacting domains.
Magnified contact map from Figure 4 clearly shows the zones where contacts are depleted by localized cohesin rings, however, this did not result in formation of self-interacting domains.

Video S1.
Video showing an intra-domain passage analysed in Figure 3.

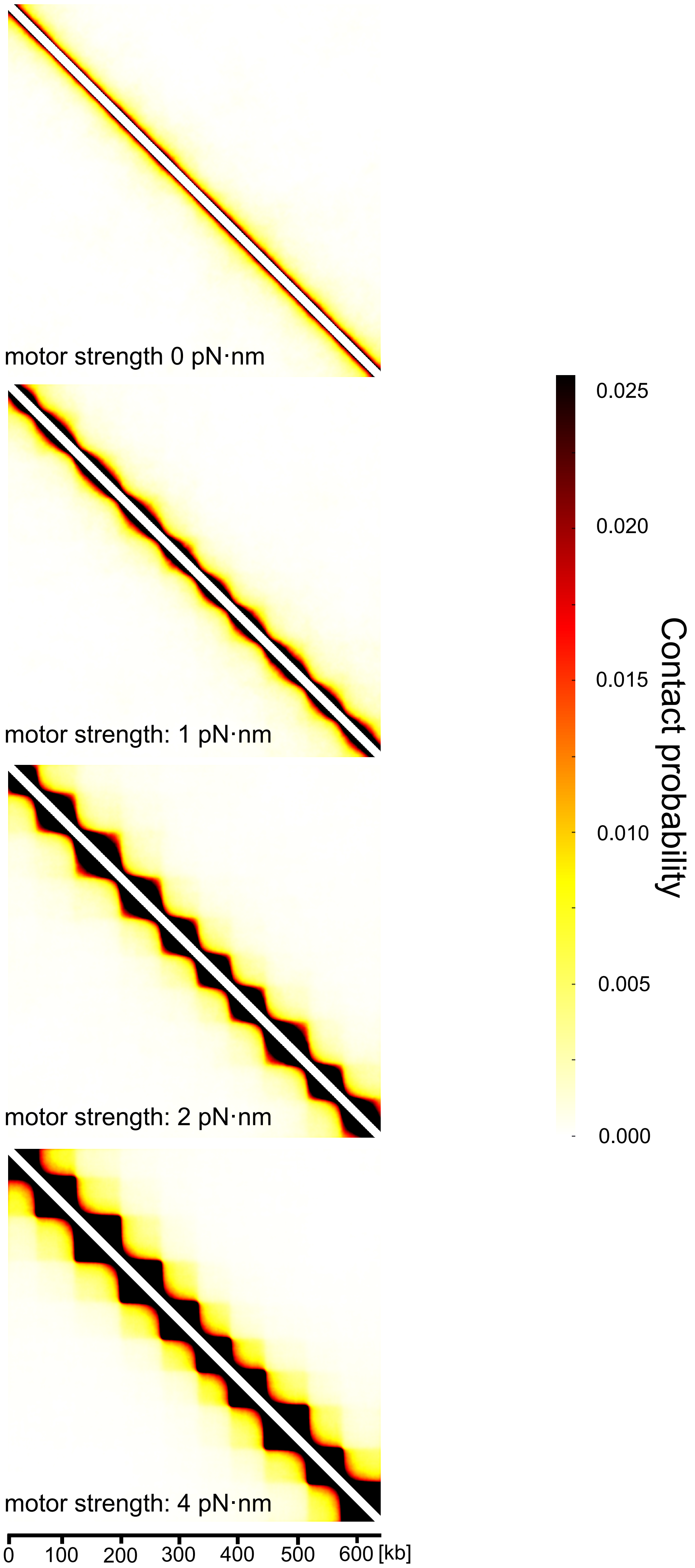
motor strength 0 pN·nm
motor strength: 1 pN·nm
motor strength: 2 pN·nm
motor strength: 4 pN·nm
0
100
200
300
400
500
600
[kb]
0.025
0.020
0.015
0.010
0.005
0.000
Contact probability

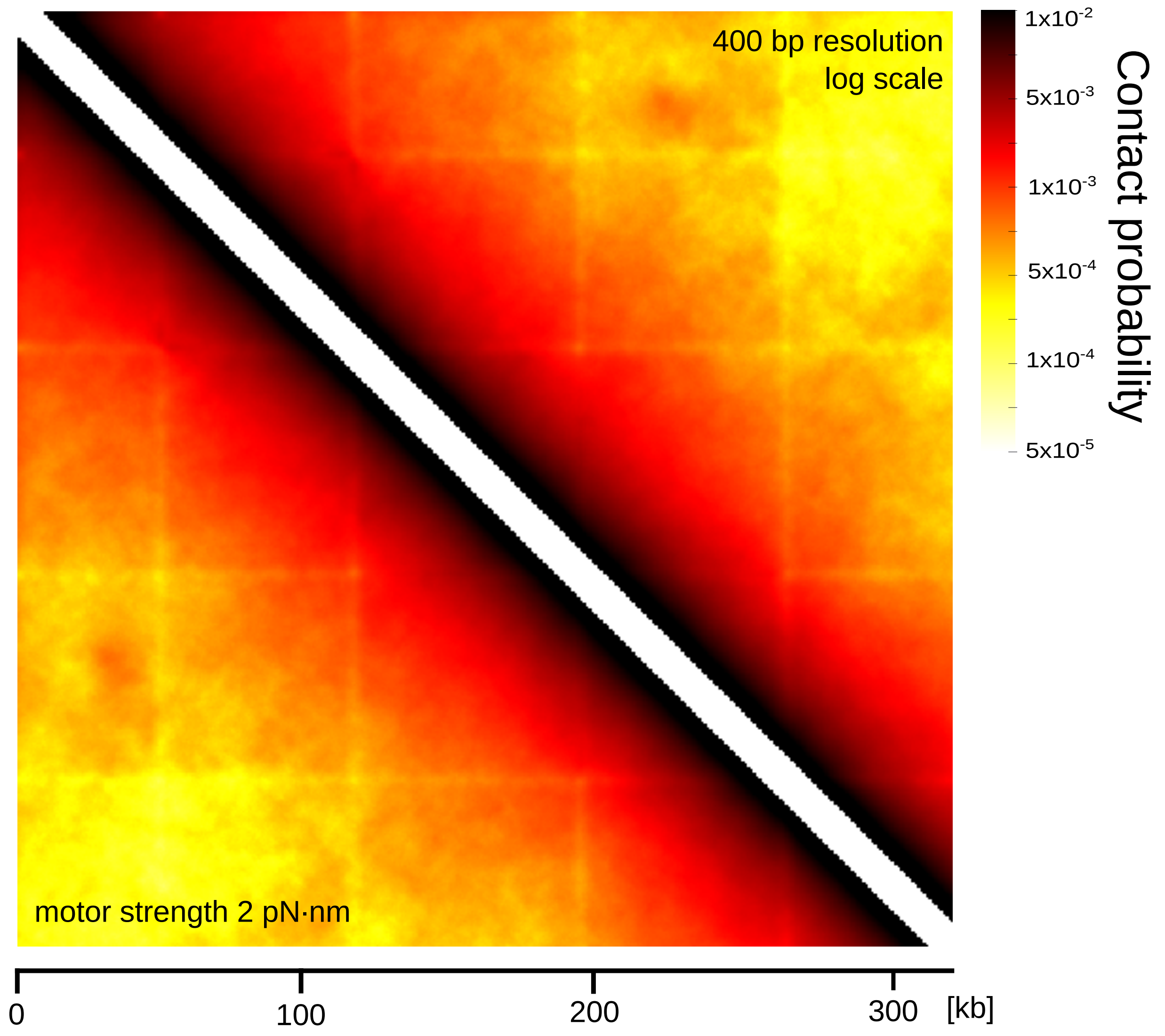

400 bp resolution
log scale
motor strength 2 pN·nm
1x10-2
5x10-3
1x10-3
5x10-4
1x10-4
5x10-5
Contact probability
0
100
200
300
[kb]
